# Impacts of dry swing intervention on bat speed and attack angle: an analysis of core intervention factors

**DOI:** 10.3389/fspor.2025.1591520

**Published:** 2025-06-18

**Authors:** Hanyao Li, Gang Cheng, Tianfeng Zhang

**Affiliations:** ^1^ School of Physical Education, Nanjing Tech. University, Nanjing, Jiangsu, China; ^2^ Sports Education and Training Science, Beijing Sport University, Beijing, China

**Keywords:** dry swing intervention, bat speed, attack angle, intervention factor, machine learning

## Abstract

**Introduction:**

This study investigated the effects of dry swing intervention using differently weighted baseball bats on bat speed and attack angles during actual swing, simulating warm-up routines. Additionally, it explored core kinematic factors impacting subsequent bat speed and attack angles.

**Methods:**

Sixty-nine baseball players were allocated by stratified randomization into three groups—normal-weight, weight, and reduced-weight—within their respective age categories. Bat swing kinematics were collected using BLAST, while bodily kinematics were captured with Rebocap sensors. Differences between pre- and post-tests were analyzed, and core intervention factors were identified with an XGBoost model and SHAP-based additive explanations.

**Results:**

No significant bat speed differences were found, but attack angles varied significantly in the normal-weight bat group for 12–14 year-olds (*p* = 0.027, ES = −0.315) and university players (*p* = 0.018, ES = 0.456). Core kinematic indicators included hip internal rotation (*p* = 0.007, ES = 0.990) and inclination angle (*p* = 0.023, ES = 0.184) showed significant differences, including and for the 12–14 age group using normal-weight bats, and hip external rotation (*p* = 0.045, ES = 1.619) for the 14–16 age group using weighted bats.

**Discussion:**

Post-test attack angles were impacted by intervention elevation and inclination angles, particularly for non-long-term bats. Adolescent athletes with shorter training term should avoid weight or reduced-weight bats for warm-up swings.

## 1 Introduction

Baseball batting constitutes a highly complex kinematic chain (1), with the primary objective of maximizing the projectile distance of the baseball through optimal kinetic transfer, initial speed controlling, bat-ball impact angle, and contact point. To achieve this, they are critical determinants. The bat speed at impact determines the baseball's initial flight velocity, while both bat-ball impact angle and contact point determines the angle of intersection between the baseball's trajectory and the ground. Baseballs travel the greatest distances only with the best combination of initial speed and intersection angle. Before executing actual swing, most players perform warm-up swings with a bat of specific weight to optimize their batting quality. During batting, the batter faces a baseball moving at an average speed of over 140 km/h (approximately 38.89 m/s) along a trajectory akin to a straight line, with merely 0.4 to 0.5 s between the moment the pitcher throws the ball and the moment the batter bats it. The batter has only 0.2 s to finish the swing, leaving only 0.2–0.3 s to decide whether to swing and how to bat (2). A batter who can improve their bat speed will have more time for decision-making. Sufficient decision-making time ensures the flexible implementation of in-game tactics and precise judgment of the contact point. Previous research has established a close association between the error in a tactical and actual contact point with body stability, which is helpful for accurately tracking the trajectory of the baseball (3), thus minimizing the deviation of an attack angle and the error between the intended and actual contact points. Although controlling the contact point can theoretically ensure that the ball follows a parabolic trajectory with an optimal initial angle to achieve maximum distance, implementing this strategy in practice is challenging. Thus, our experimental study examined the impact of using normal-weight, weight, and reduced-weight bats for dry swing intervention on the bat speed and stability of actual swing among athletes of different ages.

Bat speed and body stability are influenced by athletes' kinematic parameters and biomechanical indicators. Previous studies (4–7) have explored the effects of dry swing intervention with normal-weight, weight, and reduced-weight bats on bat speed and swing trajectory from a kinematics perspective. These studies found that among adult athletes, the use of differently weighted bats for warm-up swings had no significant impact on bat speed in pre- and post-training tests (6, 7). However, Montoya et al. (4) found that performing multiple warm-up swings using a reduced-weight or normal-weight bat improved bat speed. Furthermore, quantitative and qualitative analyses have shown that the moment of inertia (MOI) is the largest with a weighted bat, resulting in the most evident changes in an athlete's swing pattern after a dry swing intervention. However, compared to normal-weight and reduced-weight bats, the resultant change in bat speed is the smallest (5). Additionally, significant correlations have been identified between intervened bat speed (IBS), training years (TY), height (H), weight (W), and bat speed, with heavier weight contributing to faster bat speed. Studies (8–14) have also investigated the relationship between trunk rotation and force-velocity generation from a biomechanical perspective. These studies found that swing and batting rely on the biomechanical accuracy of the hips, spine, and trunk. Maximizing ground reaction force is a critical determinant of bat speed (11) hinges on effective force transmission from the pelvis to the spine (12). Moreover, the rotation of proximal body segments and force-velocity generation are fundamental to the kinetic chain of trunk rotational movements (8, 9, 14). Therefore, the hips and spine, as the most critical proximal segments, serve as key kinematic parameters for generating maximum bat speed. Other studies have employed machine learning algorithms from the field of artificial intelligence to efficiently identify potential patterns based on kinematic data (15, 16, 29–31). In summary, most studies on the effects of dry swing intervention using differently weighted bats have focused on adult athletes, such as college, university, or professional players. These studies emphasized technical routines to enhance athletes' strength and improve bat speed, whereas fewer studies have focused on adolescent athletes. Moreover, little research has combined analyses of bat speed and body stability.

To address this deficiency, the present experimental study recruited 69 athletes including junior and senior high school students, university players, and professional players. High-precision kinematic measurements of bat and body kinematics were acquired during dry swing intervention employing three types of bats: normal-weight, weight, and reduced-weight. To determine whether the dry swing intervention significantly changed bat speed and stability, the researchers analyzed the impact of dry swing intervention using differently weighted bats on actual swing among athletes in different age groups from the perspectives of mathematical statistics and machine learning. The core intervention factors affecting actual swing were identified using a machine learning model developed based on the well-known and integrated algorithm XGBoost. The results revealed the relationships between bat speed and individual dry swing intervention factors. A comparative analysis of athletes' data across age groups was conducted to investigate the profound influence of dry swing intervention using differently weighted bats on adolescent athletes in particular. Given that the results regarding athletes’ body stability were integrated into the data concerning the moment of batting, attack angle (AA) was selected to represent athletes' body stability.

## 2 Methods

### 2.1 Participants

The study participants consisted of 69 male athletes stratified into three competitive tiers: 39 adolescent athletes (junior/senior high school students), 15 collegiate athletes, and 15 professional players. The junior and senior high schoolers were recruited from Dongbeitang Middle School in Wuxi, Jiangsu Province; among whom six and nine were accredited first- and second-grade athletes, respectively. The athletes were further separated into three age groups: 12–14, 14–15, and 16–18 years. The university athletes were recruited from Nanjing Tech. University, including two accredited master sportsmen and three and ten accredited first- and second-grade athletes, respectively. The professional athletes were recruited from the Jiangsu provincial team, all of whom were accredited master sportsmen; three also served on the national team, and five were national youth team members. Table 1 lists the athletes' basic information. Among the 69 participants, 14 and 54 were left- and right-handed, respectively. All athletes participated in the study voluntarily with permission from their managers. Prior to recruitment, individual conversations were held with the athletes regarding their injury history, current status, training experience, and involvement in sports other than baseball. This information was used to determine whether candidates met the experiment's inclusion criteria, which were as follows: (a) baseball must be the athlete's only competitive sport; (b) non upper-limb or trunk musculoskeletal injuries within the previous four months; (c) non shoulder, elbow, or spinal surgery in the past six months; (d) non usage of medications or supplements that could influence athletic performance; and (e) willingness to complete all testing sessions. During recruitment, each athlete got a thorough explanation of the experimental procedures, potential risks, and anticipated benefits. Written informed consent was obtained from all participants before data collection.

**Table 1:** Basic information of Athletes.

Groups	Age (Y) ($\bar{x}\pm s$)	Height (CM) ($\bar{x}\pm s$)	Weight (KG) ($\bar{x}\pm s$)	Training years (Y) ($\bar{x}\pm s$)	Count
12–14 age	12.25 ± 0.31	157.67 ± 2.13	49.21 ± 3.34	1.33 ± 0.26	12(L:1; R:11)
14–15 age	14.33 ± 0.14	173.33 ± 1.61	61.25 ± 2.24	4.00 ± 0.56	12(L:4; R:8)
16–18 age	16.38 ± 0.18	178.23 ± 1.50	71.23 ± 2.48	5.15 ± 0.36	15(L:3; R:12)
U-Athletes	20.00 ± 0.28	182.47 ± 1.65	80.83 ± 2.61	7.27 ± 0.55	15(L:3; R:12)
Pro-Athletes	22.53 ± 0.48	182.13 ± 1.37	82.40 ± 3.56	10.27 ± 0.33	15(L:3; R:12)

U- Athletes denotes university Athletes; Pro- Athletes denotes professional Athletes; L denotes left-handed batter; R denotes right-handed batter.

### 2.2 Experimental design

Consistent with age classifications for competitive baseball in terms of the rules published by the General Administration of Sport of China, experimental schemes were implemented using age-appropriate equipment: for the adolescent athletes, soft bats for the 11–13 age group and metal bats for the 16–18 cohort. For the adult athletes, collegiate athletes utilized metal bats, while professionals employed regulation wooden bats. The bats used for the experiment were differently weighted bats typically used in regular athletic events (see Table 2). To ensure similar performance levels among groups within the same age cohort, we employed the skill of stratified randomization (32). For participants aged 16 years and older who held athlete-grade certificates, we first stratified them into three tiers, “Master Sportsman”, “First-Grade Athlete”, and “Second-Grade Athlete”. Athletes in each tier were then randomly allocated into one of three groups—normal-weight, weight, or reduced-weight. For the 12–13 year-old and 14–15 year-old cohorts, who had not yet obtained athlete-grade certificates, stratification was based on years of formal training, followed by random assignment. To further minimize bias arising from prior experience, the allocation for 12–15-year-olds also considered current skill level, height, and body mass. In addition, we actively balanced handedness to guarantee that each experiment group contained at least one left-handed batter. Standard baseballs weighing 142 grams were used for testing. One week prior to the experiment, participants were instructed to avoid consuming alcohol, overeating, or staying up late. They were also prohibited from consuming caffeinated products or any other substances that might enhance athletic performance within 48 h of the experiment. On the day of the experiment, participants refrained from engaging in additional physical activities beyond regular training sessions. Furthermore, they were required to ensure they had received at least eight hours of sleep the night before the experiment.

**Table 2:** Information on bats used in each group.

Groups	Normal-weight bat	Weight bat	Reduced-weight bat
12–14 age	EASTON-DMEN (78CM;566G)	SSK-SUPER CONDER (83CM;850G)	Self-made (80CM;300G)
14–15 age	SSK-SUPER CONDER (83CM;850G)	SSK with Ring (83CM;1350G)	EASTON-ELEVET (76.2CM;550G)
16–18 age	SSK-SUPER CONDER (83CM;850G)	SSK with Ring (83CM;1350G)	MIZUNO-FUNGO (91CM;530G)
U-Athletes	ASICS TN600 (83CM;870G)	ASICS with Ring (83CM;1400G)	MIZUNO-FUNGO (91CM;530G)
Pro- Athletes	YANASE-PRO (84CM;920G)	MARUCCI-PRO MAPLE (84CM;1300G)	MIZUNO-FUNGO (91CM;530G)

U- Athletes denotes university Athletes; Pro- Athletes denotes professional Athletes.

### 2.3 Experimental procedures

**Figure 1:**
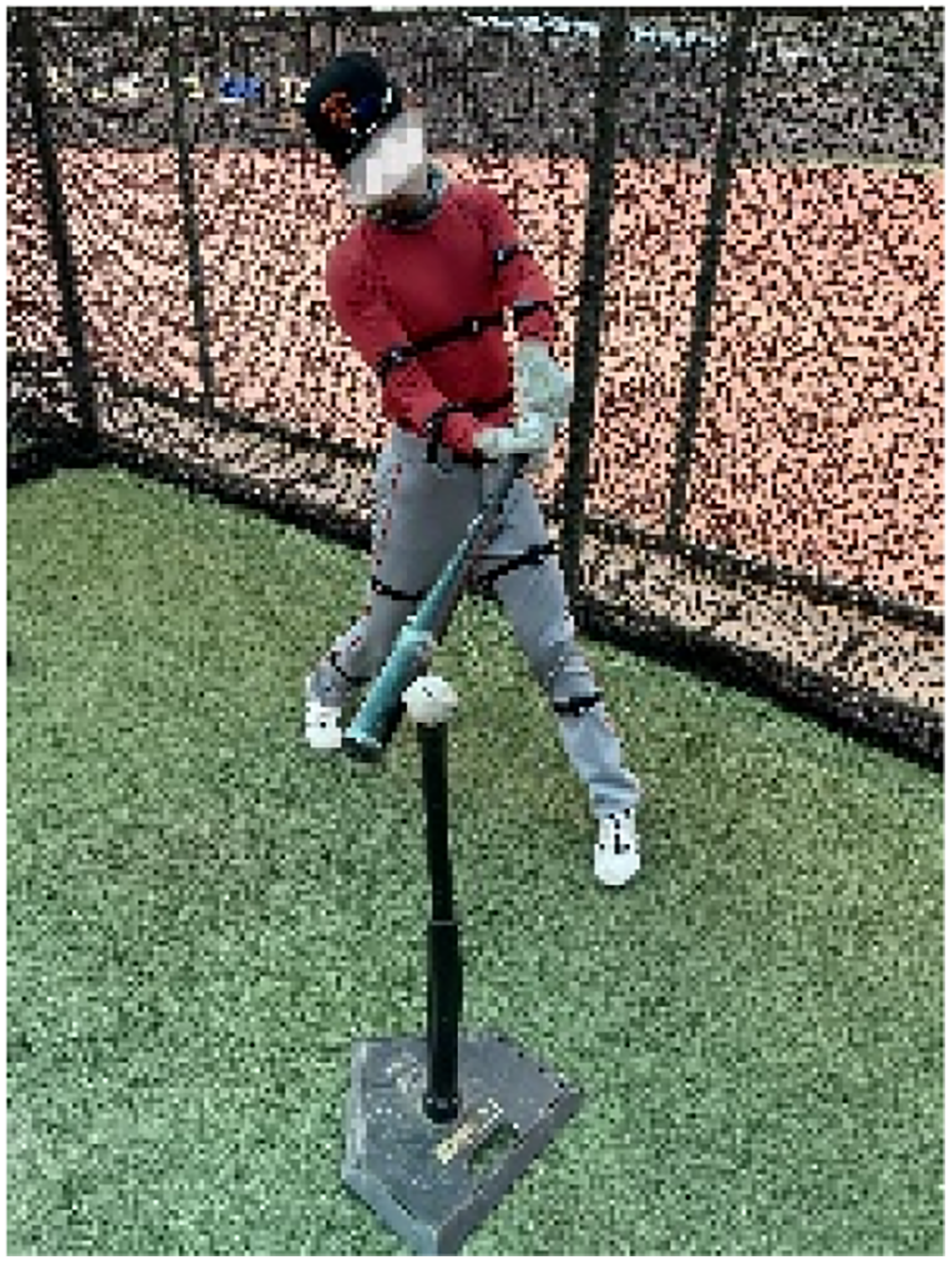
Testing of the “T” batting platform.

To ensure participants were in optimal condition for athletic performance, standardized dynamic activation exercises were conducted prior to the formal test. After these activation exercises, each athlete was equipped with a Rebocap sensor and engaged in adaptive practice using a group-specific designated bat fitted with a BLAST device (Figure 1). The athletes performed adaptive batting on a T-shaped batting platform at the testing site to ensure proper warm-up and find the optimal attack angle and suitable batting platform height. The height of the platform was adjusted according to individual athletes' preferences, except during the formal test, which commenced once the athletes indicated they were ready following their warm-up swings. During the pre-test phase, each athlete performed three official swings, with a 10-second interval between each swing. After completing the pre-test, the athletes performed five dry swings in two minutes using the assigned intervention bat. The two-minute duration was selected to allow sufficient time for the batter to walk from the warm-up zone to the batter's box. Subsequently, three post-tests were conducted, with a 10-second interval between each swing. All testing data were documented in detail and saved.

### 2.4 Data collection

#### 2.4.1 Bat kinematic data collection

**Figure 2:**
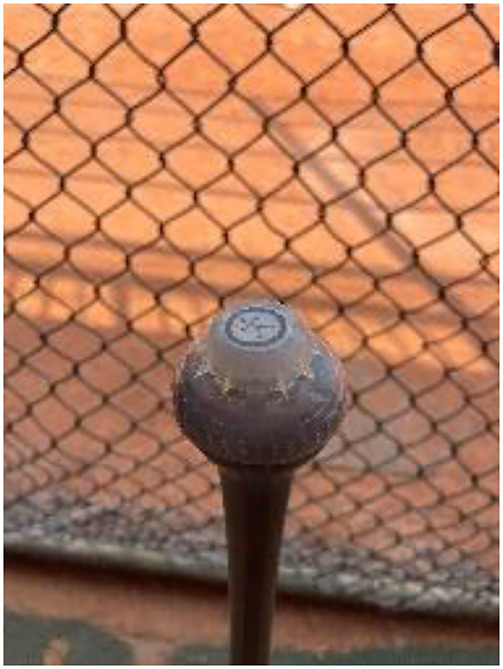
Blast device mounted on the Bat's end.

We use a BLAST (USA) device to collect bat kinematic data attached to the knob of the bat (Figure 2). The data collected included pre-test, intervention, and post-test bat speed (BS), as well as pre- and post-test rotational acceleration (RA), attack angle (AA), on-plane efficiency (OPE), early connection (EC), and connection at impact (CAI; Table 3).

**Table 3:** The interpretation of BLAST data.

Indictor	Meanings
BS	The speed of the bat at the moment during batting the ball
RA	The acceleration of the bat during batting
AA	The collision angle between the ball and the bat at the moment of hitting
OPE	The alignment of the bat with the desirable swing plane during the hitting process
EC	The angle between the bat and the vertical line of the ground during the stride phase
CAI	The angle between the bat and the vertical line of the ground at the moment of contact.

**Table 4:** The interpretation of bodily kinematic data.

Indictor	Meanings
L3IR	The third lumbar vertebra internal rotation
L3ER	The third lumbar vertebra external rotation
L3MS	The third lumbar vertebra maximum angular speed
LHIR	Lead hip internal rotation
LHER	Lead hip external rotation
LHMS	Lead hip maximum angular speed
PHIS	Push-off hip internal rotation
PHER	Push-off hip external rotation
PHMS	Push-off hip maximum angular speed
Q	The elevation angle of the body on the Z-axis when batting the ball (Inclination angle)
Y	The elevation angle of the body on the *Y*-axis when batting the ball (Elevation angle)
I-XXX	Abbreviated prefix denoting the intervention segment targeting specific indicators

For right-handed hitters, the lead hip corresponds to the left hip, and the push-off hip corresponds to the right hip; vice versa for left-handed hitters.

#### 2.4.2 Bodily kinematic data collection

**Figure 3:**
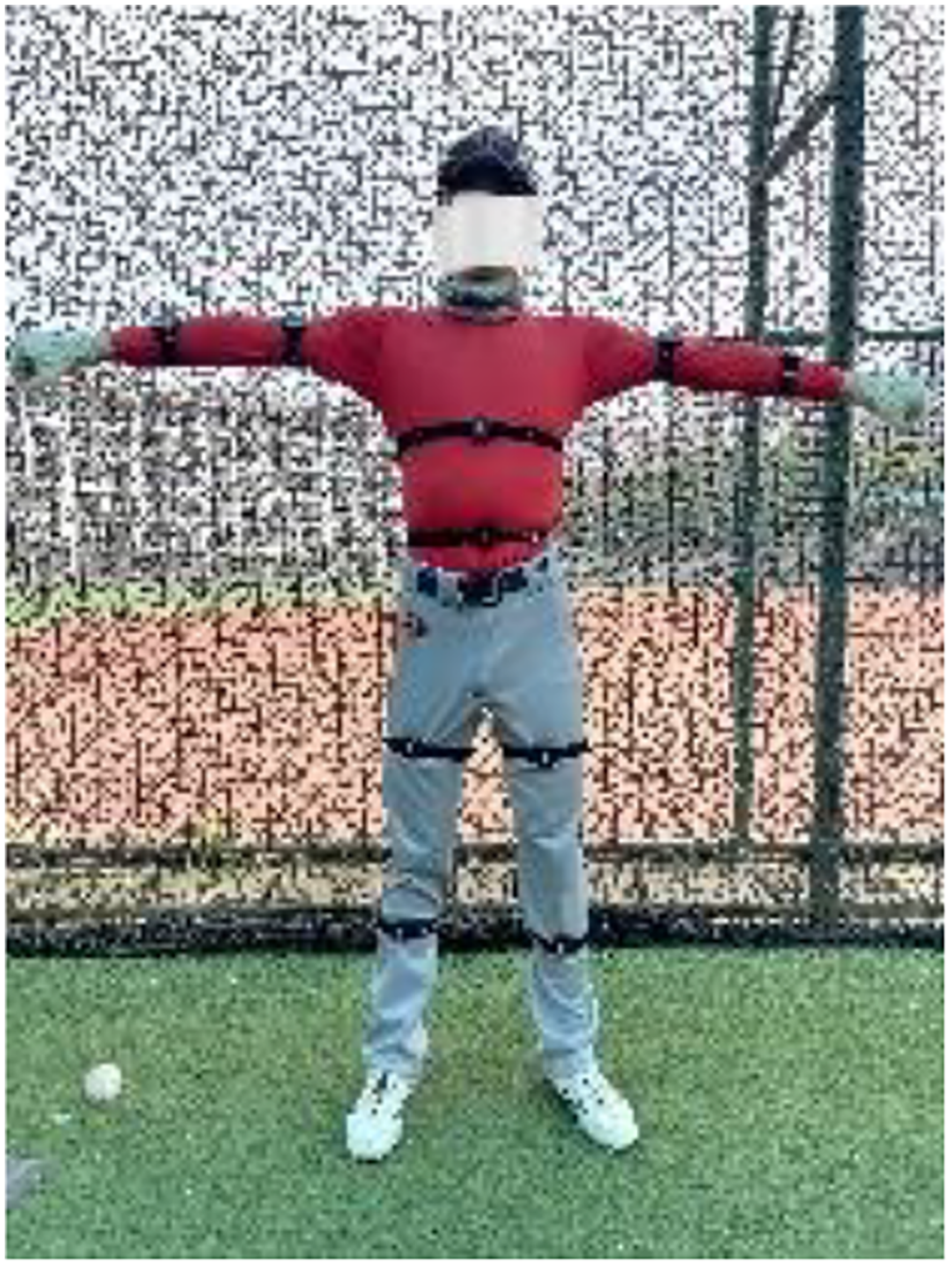
Sensor device on the Athlete's body.

**Figure 4:**
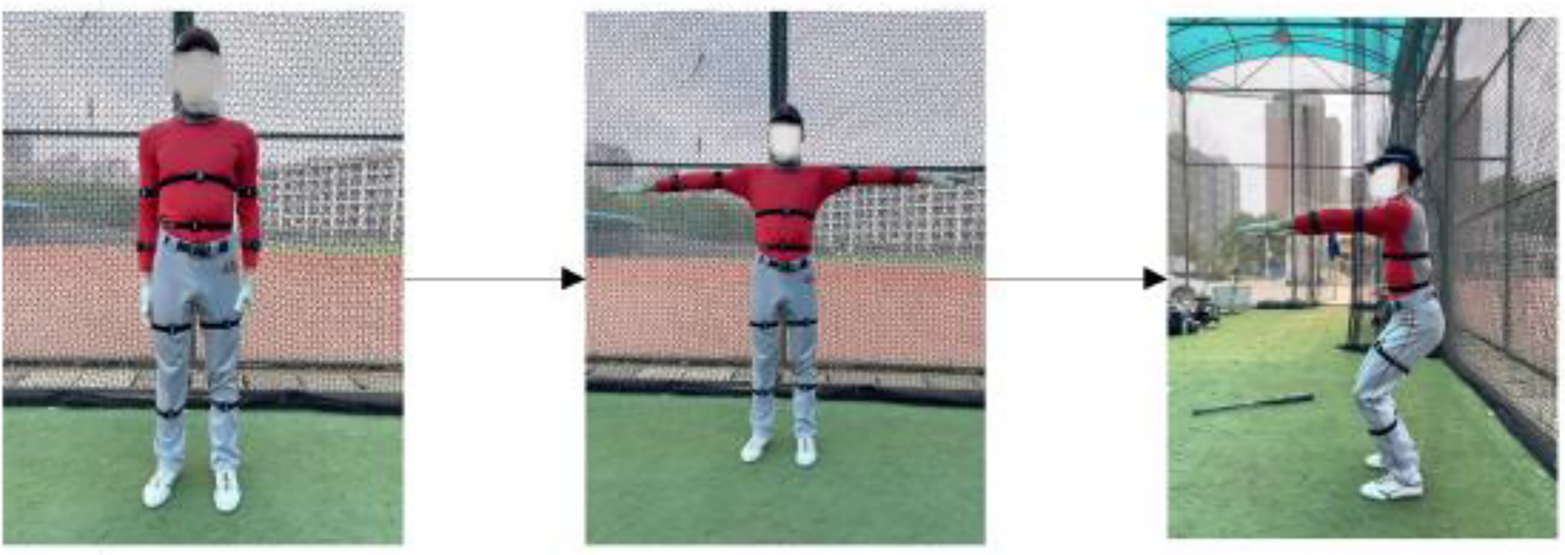
Motion calibration (A-T-S) before data collection to improve accuracy.

**Figure 5:**
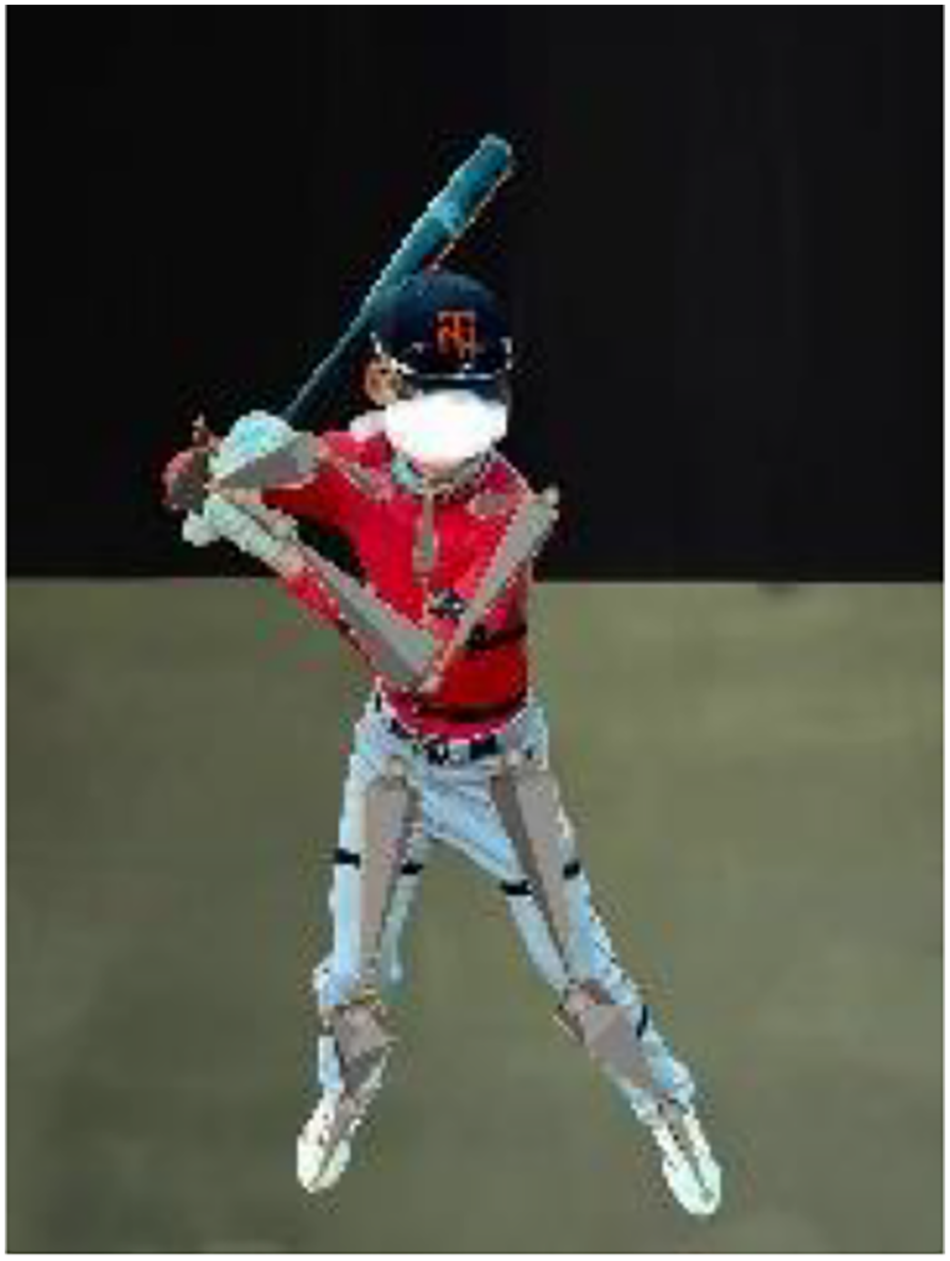
Sensor collecting and generating 3D biometric skeletal models during each Athlete's swing.

Athletes' bodily kinematic data were collected using 10 IMU Rebocap sensors (China) [33] positioned at the xiphoid process, the midpoint of the left and right humeri, the midpoint of the left and right radii, the anterior of the third lumbar vertebra, the midpoint of the left and right femora, and the midpoint of the left and right tibiae (Figure 3). The sensors' height settings were adjusted according to each athlete's height. To ensure the accuracy of the athletes' skeletal models, calibration was performed prior to motion capture using three distinct poses: A, T, and S (Figure 4). The raw data for sensor position and orientation were independently filtered along each global axis using a fourth-order Butterworth filter with a cutoff frequency of 20 Hz (17). This filtering method effectively removed high-frequency noise, ensuring the data's smoothness and accuracy. Motion capture was performed on athletes' pre-test, intervention, and post-test batting, and the generated skeletal models were exported (Figure 5). Data from the two core coordinate systems, the pelvis (Pe) and spine (Sp), were collected to quantify the athletes' core kinematic parameters. The Pe coordinate system used the two positions of lead hip (LH) and push-off hip (PH), and the Sp system used the third lumbar vertebra (L3) position. Rotation was defined as the axial rotation of the spine and pelvis. Three-dimensional Euler angles (using the Z-Y'-X’’ rotation sequence) were employed to quantify the internal and external rotation of L3, LH, and PH (Y′ rotation), as well as the tilt angles of L3 in terms of elevation (Z’ rotation) and lateral flexion (X' rotation). Internal rotation of the spine and pelvis was defined as the clockwise rotation angle from the standing preparation phase to the extension phase. External rotation was defined as the maximum counterclockwise rotation angle from the end of internal rotation to the completion of the batting motion, measured in radian. Anterior–posterior tilt and lateral flexion angles were measured as the difference between the static body Z and *Y* axis angles at the moment of batting and in the preparation phase, in radian. These angles were primarily used for data visualization and were not directly included in calculations. Kinematic velocity metrics were represented as the maximum angular speed.

### 2.5 Statistical analysis

To facilitate data processing, we saved the data collected using BLAST in Excel spreadsheets. The data were categorized by age group and bat weight. The descriptive statistical results are represented by ($\bar{x}\pm s$) and [M(P25,P75)]. We used the Shapiro–Wilk test in SPSS Statistics 26.0 (USA) to conduct a normality test (*p* > 0.05). For data with a normal distribution, a paired samples t-test was conducted to compare changes in indicators before and after dry swing intervention. Data without a normal distribution underwent a Mann–Whitney *U*-test. We used Python (USA) to calculate Cohen's d effect size (ES) for the interventions. To correct for the overestimation of effect sizes in small datasets, the Hedges' g correction factor J (Equation 1) was applied to the Cohen's d values. The resulting effect sizes were stored in an Excel spreadsheet.

(1) $$J=1-\frac{3}{4\;*\;(n_{1}+n_{2}-2)-1}$$

where $n_{1}$ and $n_{2}$ denote the size of the pre-test and post-test samples, respectively.

We categorized the intervention effects based on effect size thresholds defined by G*Power: ES < 0.10 was classified as no effect, 0.10 ≤ ES < 0.30 as a low effect, 0.30 ≤ ES < 0.50 as a moderate effect, and ES > 0.50 as a high effect (18).

### 2.6 Intervention factors selection model

XGBoost (eXtreme Gradient Boosting) is an efficient machine learning algorithm based on the gradient boosting strategy which is widely applied for feature selection and regression prediction tasks. Its core scheme is to continuously optimize the model through gradient boosting methods based on decision trees, guiding it closer to the optimization solution step by step. During the training process, XGBoost optimizes the model by minimizing the objective function, which consists of a loss function and a regularization term, as shown in Equation (2).

(2) $$\tau (\theta )=\sum_{i=1}^{n}l(y_{i},{\hat{y}}_{i})+\sum_{k=1}^{K}\Omega (f_{k})$$

where the loss function $l(y_{i},{\hat{y}}_{i})$ can be taken as either Mean Squared Error (MSE) or Log Loss, to measure the error between the model's predicted value ${\hat{y}}_{i}$ and the real value $y_{i}$.

Since this issue-studying in this paper is more inclined towards regression analysis, in Equation (2), the mean squared error is employed as the loss function, and shown as equation (3),

(3) $$l(y_{i},{\hat{y}}_{i})=(y_{i}-{\hat{y}}_{i}{)}^{2}$$

In Equation (2), $\Omega (f_{k})$ represents the regularization term of the model, which works to control the complexity of the trees to prevent overfitting. Wherein, the reg_alpha parameter is a key hyper-parameter that controls the strength of L1 regularization and also play a key role in the feature selection process. L1 regularization is shown in Equation (4),

(4) $$\Omega_{\alpha }(f)=\mathrm{reg}\_\mathrm{alpha}\sum \left|w_{j}\right|$$

With comparison of L2 regularization, L1 regularization has the characteristic of sparsity, which can drive some feature weights to converge to zero. This allows the model to automatically select the most representative features and enhance its generalization ability. In practice, appropriately increasing its value of the reg_alpha parameter can degrade the interference of unimportant features and improve the model's adaptability to unknown data. This paper takes the average of its empirical values of the reg_alpha parameter after several rounds of supervised learning training.

Baseball batting entails non-linear motion. Thus, this study focused on XGBoost-based variable selection models for intervention factors. Models 1–3 were established to select the intervention factors of post-test bat speed (HBS) following the use of normal-weight, weight, and reduced-weight bats, respectively. Models 4–6 were constructed to select post-test attack angles following the use of normal-weight, weight, and reduced-weight bats, respectively.

The output variables of Models 1–3 were post-test bat speed (HBS) after dry swing intervention using three differently weighted bats. The input features included athletes' years of training (TY), height (H), weight (W), IBS, intervened third lumbar vertebra internal rotation (IL3IR), intervened third lumbar vertebra external rotation (IL3ER), intervened third lumbar vertebra maximum angular speed (IL3MS), intervention-led hip internal rotation (ILHIR), intervention-led hip external rotation (ILHER), intervention-led hip maximum angular speed (ILHMS), intervened push-off hip internal rotation (IPHIS), intervened push-off hip external rotation (IPHER), and intervened push-off hip maximum angular speed (IPHMS). Models 4–6 predicted post-intervention attack angles (HAA) after the use of the three differently weighted bats. These models’ input features included athletes' years of training (TY), height (H), weight (W), intervention inclination angle (IQ), intervention elevation angle (IY), intervention early connection (HEC), intervention connection at impact (HCAI), and intervention on-plane efficiency (HOPE) (see Table 4). We conducted intervention factor selection modeling using a custom program obtained from the Python Scikit-learn library. To further improve the models' generalization ability, we conducted random sampling on the raw samples and separated them into training and testing data sets at a seven-to-three ratio. The training data set was used for model training, tuning, and parameter optimization, while the testing data set was used for model testing and validation. Model tuning and parameter optimization were implemented using GridSearchCV. The key hyper-parameters optimized in this study include max_depth [5, 15], n_estimators [50, 300], learning_rate [0.05, 0.3], subsample [0.5, 1], and reg_alpha [0.001, 10]. These parameter ranges were selected to achieve a balance between model complexity, generalization performance, and computational efficiency. The other hyper-parameters, such as colsample_bytree etc., were configured at their default values, as their impact on model performance was considered secondary ones. To guarantee optimization reliability, implementing 5-fold cross-validation, achieving an optimal balance between computational efficiency and model stability. This approach guaranteed robust generalization capability in the final model. Model testing and validation were measured using the mean test score (MTS), which facilitated evaluation of the accuracy and reliability of the feature variables the model selected. The MTS is calculated using equation (5),

(5) $$MTS=\frac{1}{n}\sum_{i=1}^{n}S_{i}$$

where *n* represents the number of folds in cross-validation, and $S_{i}$ denotes the test score of the i-th fold cross-validation.

Additionally, we used the Shapley additive explanation (SHAP) algorithm to explain the variables selected by the model. For a model with *n* features, the calculation of its Shapley value $\phi_{i}$ for i-th feature is based on cooperative game theory, as shown in Equation (6),

(6) $$\phi_{i}=\sum_{S\subset N,i\in S}\frac{(|S|-1)!(n-|S|)!}{n!}[v(S)-v(S-\{i\})]$$

where *N* is the set of all features, *S* is a subset containing some features, |S| represents the size of *S* subset, and v(S) is the model's predicted value corresponding to the feature subset *S*.

## 3 Results

### 3.1 Individual intervention groups' paired *t*-test results

**Figure 6:**
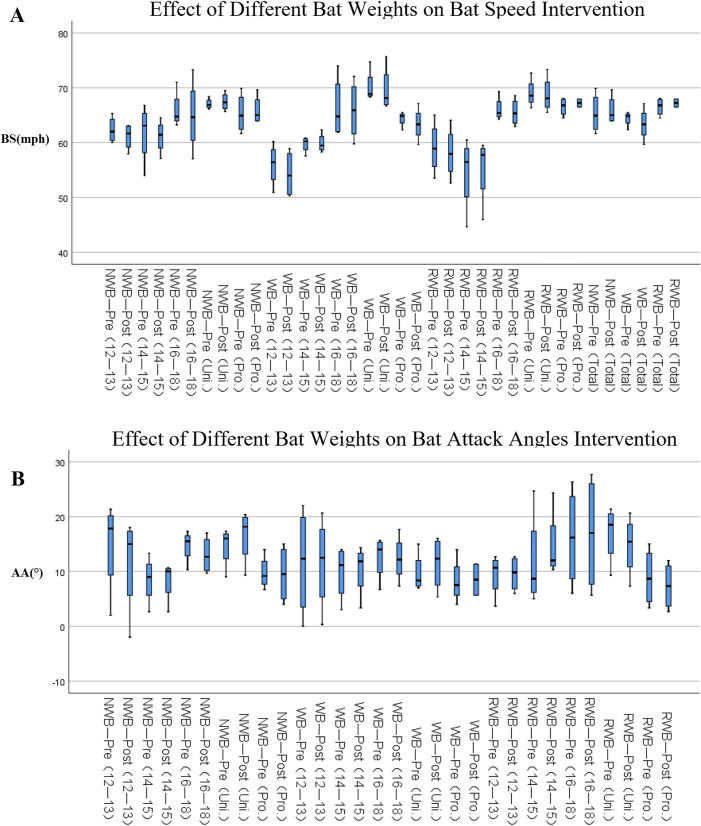
Box plot representing the data of the swinging motion of Bat speed **(A)** and attack angles **(B)** as collected by the blast. Box boundaries and the horizontal lines represent interquartile ranges Q1–Q3 and mean measurements, respectively. Whiskers above and below the boxes mark the maximum and minimum values, respectively.

Paired samples *t*-tests were conducted using athletes' pre- and post-test bat and body kinematic data across age groups to observe the differences between the pre- and post-test results. Tables 5–7 present the results of the paired *t*-test analysis for significance. Table 5 shows that the paired *t*-tests of all bat speed indices indicated no significant differences between the pre- and post-tests (Figure 6A). Table 6 shows that the age 12–14 and university intervention groups using normal-weight bats exhibited significant differences in AA (*p* = 0.027, ES = 0.315; *p* = 0.018, ES = 0.456) (Figure 6B). Table 7 shows that the age 16–18 intervention group using weight bats exhibited significant differences in RA (*p* = 0.047, ES = 0.173), the corresponding group using normal-weight bats demonstrated significant differences in OPE (*p* = 0.037, ES = −1.235), and their counterparts using weight bats presented significant differences in EC (*p* = 0.02, ES = 0.280), whereas the university intervention group in the same age bracket using reduced-weight bats exhibited significant differences in CAI (*p* = 0.049, ES = 0.290). The age 12–14 intervention group using normal-weight bats showed significant differences in LHIR (*p* = 0.007, ES = 0.99) and Q (*p* = 0.023, ES = 0.184), and the age 14–16 intervention group using weight bats demonstrated significant differences in LHER (*p* = 0.045, ES = 1.619), with no significant differences observed in other indicators.

**Table 5:** Effect of different bat weights on Bat speed intervention: average test values.

Types of Bats Intervention	Groups	Pre-test (Q) ($\bar{x}\pm s$)	Post-test (H) ($\bar{x}\pm s$)	*p*-Value	Hedges’g
N-weight Bat	12–14	62.33 ± 1.20	61.08 ± 1.21	0.099	−0.448
	14–15	61.74 ± 5.48	61.11 ± 1.54	0.668	−0.123
	16–18	65.29 ± 1.48	64.58 ± 2.60	0.652	−0.254
	Uni.	68.13 ± 1.12	68.78 ± 1.47	0.528	0.204
	Pro.	67.65 ± 2.71	68.17 ± 2.50	0.371	0.081
	Total	65.29 ± 0.97	65.06 ± 1.08	0.625	−0.055
Weight Bat	12–14	55.98 ± 1.94	54.27 ± 2.19	0.263	−0.359
	14–15	59.71 ± 0.75	59.88 ± 0.88	0.830	0.093
	16–18	65.77 ± 2.27	65.41 ± 2.15	0.634	−0.066
	Uni.	69.27 ± 1.50	68.85 ± 1.79	0.530	−0.102
	Pro.	64.96 ± 0.81	64.21 ± 1.46	0.377	−0.256
	Total	63.60 ± 1.77	63.00 ± 1.27	0.101	−0.100
R-weight Bat	12–14	58.27 ± 2.96	58.13 ± 2.37	0.882	−0.023
	14–15	54.49 ± 3.44	55.23 ± 3.14	0.300	0.098
	16–18	66.17 ± 0.86	65.97 ± 1.07	0.764	−0.082
	Uni.	71.36 ± 2.54	71.28 ± 2.87	0.917	−0.012
	Pro.	67.12 ± 0.88	67.63 ± 0.53	0.474	0.284
	Total	64.24 ± 1.52	64.25 ± 1.52	0.958	0.002

Uni. denotes university athletes; Pro. denotes professional athletes; N-weight denotes normal-weight; R-weight denotes reduced-weight.

**Table 6:** Effect of different Bat weights on bat attack angles intervention.

Types of Bats Intervention	Groups	Pre-test(Q) ($\bar{x}\pm s$)	Post-test(H) ($\bar{x}\pm s$)	*p*-Value	Hedges’g
N-weight Bat	12–14	14.75 ± 4.36	11.50 ± 6.61	0.027*	−0.315
	14–15	9.0 (4.2,12.3)	10 (4.4,10.6)	0.686	−0.016
	16–18	13.20 ± 1.88	12.33 ± 1.49	0.502	0.040
	Uni.	13.87 ± 1.63	15.93 ± 2.01	0.018*	0.456
	Pro.	9.47 ± 1.23	8.93 ± 2.14	0.761	0.087
Weight Bat	12–14	11.67 ± 5.01	11.50 ± 4.28	0.910	0.326
	14–15	9.83 ± 2.53	10.33 ± 2.42	0.519	0.206
	16–18	12.73 ± 1.60	12.60 ± 1.67	0.896	−0.033
	Uni.	9.93 ± 1.44	11.13 ± 1.96	0.435	0.098
	Pro.	7.7 (5.7,13.5)	11.3 (5.7,11.5)	0.841	0.456
R-weight Bat	12–14	9.42 ± 1.99	9.58 ± 1.63	0.874	0.282
	14–15	8.7 (5.6,21.0)	12.0 (10.7,21.3)	0.343	0.172
	16–18	14.33 ± 4.00	15.33 ± 4.44	0.329	−0.124
	Uni.	18.87 ± 2.84	17.51 ± 3.53	0.341	−0.016
	Pro.	9.07 ± 2.08	7.73 ± 1.75	0.230	−0.280

For data not following a normal distribution, [M(P25, P75)] is used to represent the data, with the *p*-value as the result of the Mann–Whitney *U*- test.

*Indicates a significant difference.

**Table 7:** Other significance indicators: average test values.

Groups	Indicator	Pre-test (Q) ($\bar{x}\pm s$)	Post-test (H) ($\bar{x}\pm s$)	*p*-Value	Hedges’g
12–14 N-weight Bat	LHIR	32.78 ± 10.31	43.46 ± 8.36	0.007*	0.990
	Q	10.12 ± 7.56	11.75 ± 7.89	0.023*	0.184
14–15 Weight Bat	LHER	52.43 ± 9.47	75.17 ± 14.44	0.045*	1.619
16–18 Weight Bat	RA(g)	6.99 ± 4.61	7.87 ± 4.67	0.047*	0.173
	EC(°)	91.80 ± 13.16	95.93 ± 13.54	0.020*	0.280
16–18 N-weight Bat	OPE(%)	63.20 ± 4.79	55.13 ± 6.83	0.037*	−1.235
Uni. R-weight Bat	CAI(°)	92.67 ± 6.98	90.20 ± 5.64	0.049*	−0.351

RA, the acceleration of the bat during the hitting process; EC, the angle between the bat and the vertical line of the ground during the stretching stage; OPE, the degree of consistency between the bat and the ideal batting plane during the batting process; CAI, the angle between the bat and the vertical line of the ground while batting. LHIR, guide the internal rotation angle of the buttocks; Q, the elevation angle of the body on the Z-axis when batting the ball; LHER, guide the external rotation angle of the buttocks.

*Indicates a significant difference.

### 3.2 Intervention factor selection results

**Figure 7:**
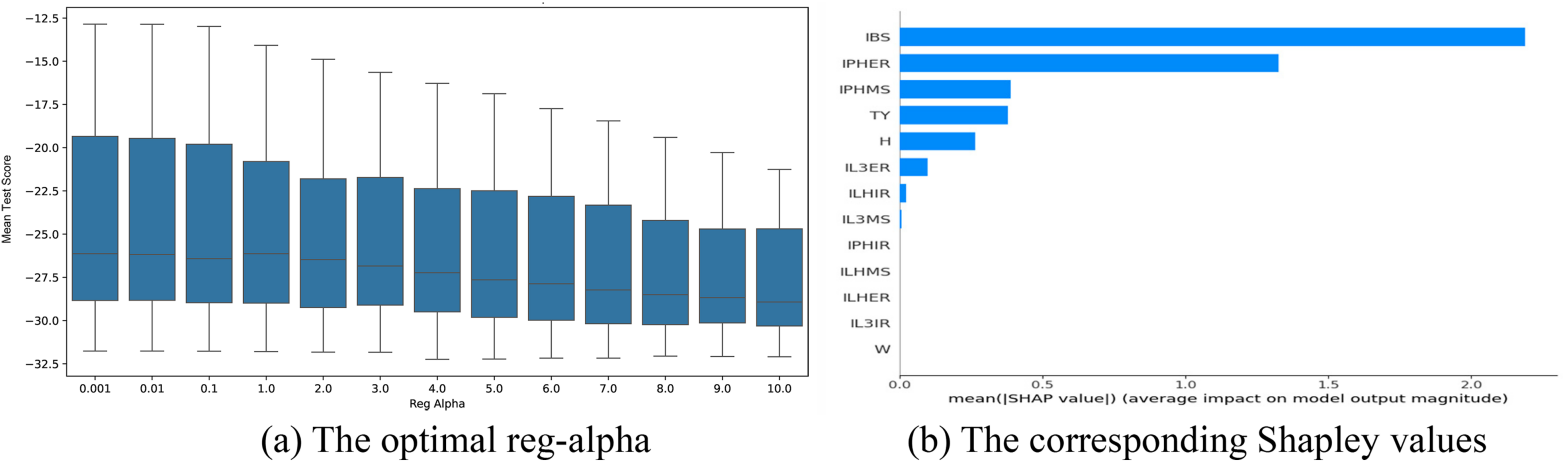
Visualization of screening results for the normal-weight bat intervention of the BS group. **(a)** The optimal reg-alpha. **(b)** The corresponding shapley values.

**Figure 8:**
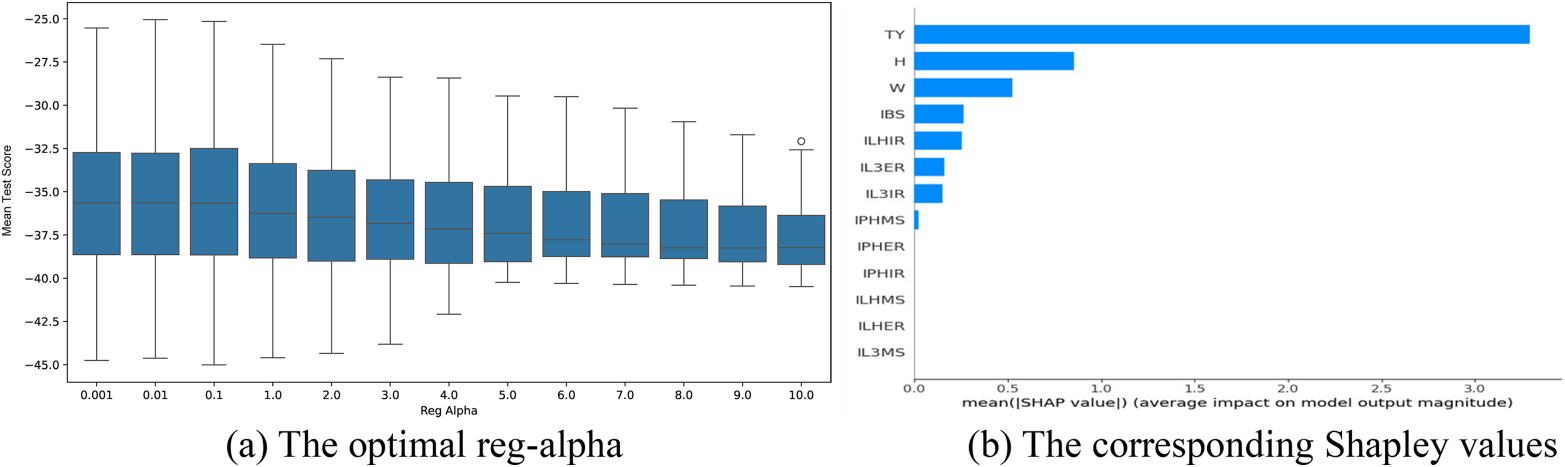
Visualization of screening results for the weight bat intervention of the BS group. **(a)** The optimal reg-alpha. **(b)** The corresponding shapley values.

**Figure 9:**
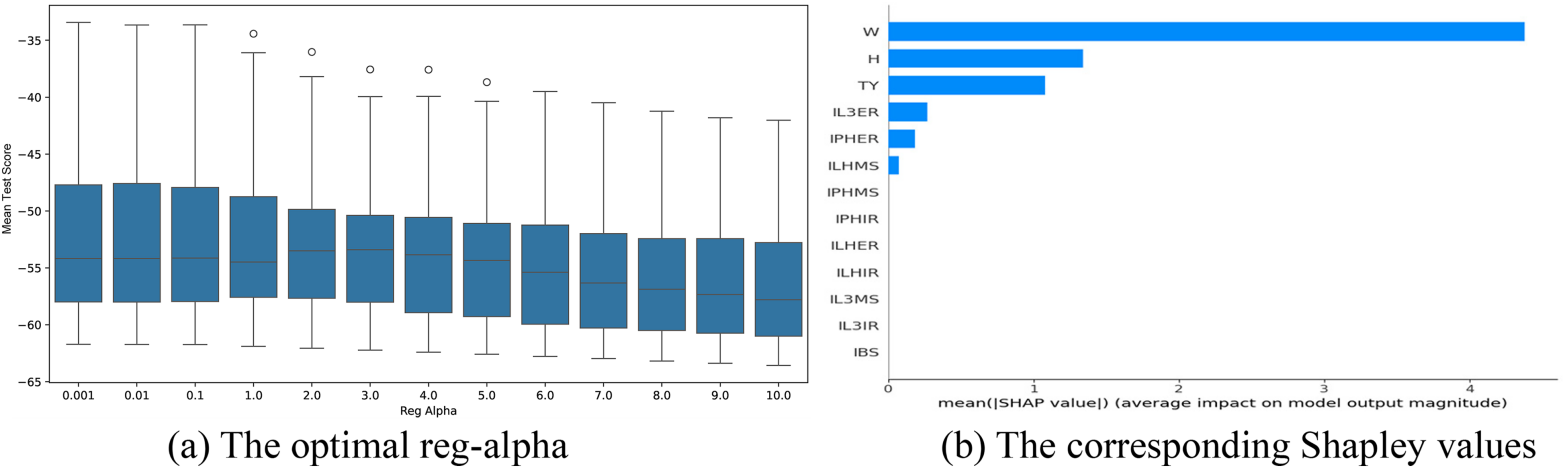
Visualization of screening results for the reduced-weight bat intervention of the BS group. **(a)** The optimal reg-alpha. **(b)** The corresponding shapley values.

The optimal parameter tuning results for reg_alpha in the bat speed intervention factors selection models based on the XGBoost algorithm are shown in Figures 7a–9a. Optimal mean test scores (MTS) for Models 1–3 were obtained when the reg_alpha parameters in these models were assigned the values 1, 0.01, and 3, respectively. The ranking results of the corresponding Sharply values are depicted in Figures 7b–9b, respectively. These illustrations show that the core indicators IPHER and IPHMS affected the post-test bat speeds of the intervention groups that used normal-weight bats (Figure 7b); ILHIR, IL3IR, and IL3ER affected the post-test bat speeds of the intervention groups that used weighted bats (Figure 8b); and IL3ER, IPHER, and ILHMS impacted the post-test bat speeds of the intervention groups that used reduced-weight bats (Figure 9b).

**Figure 10:**
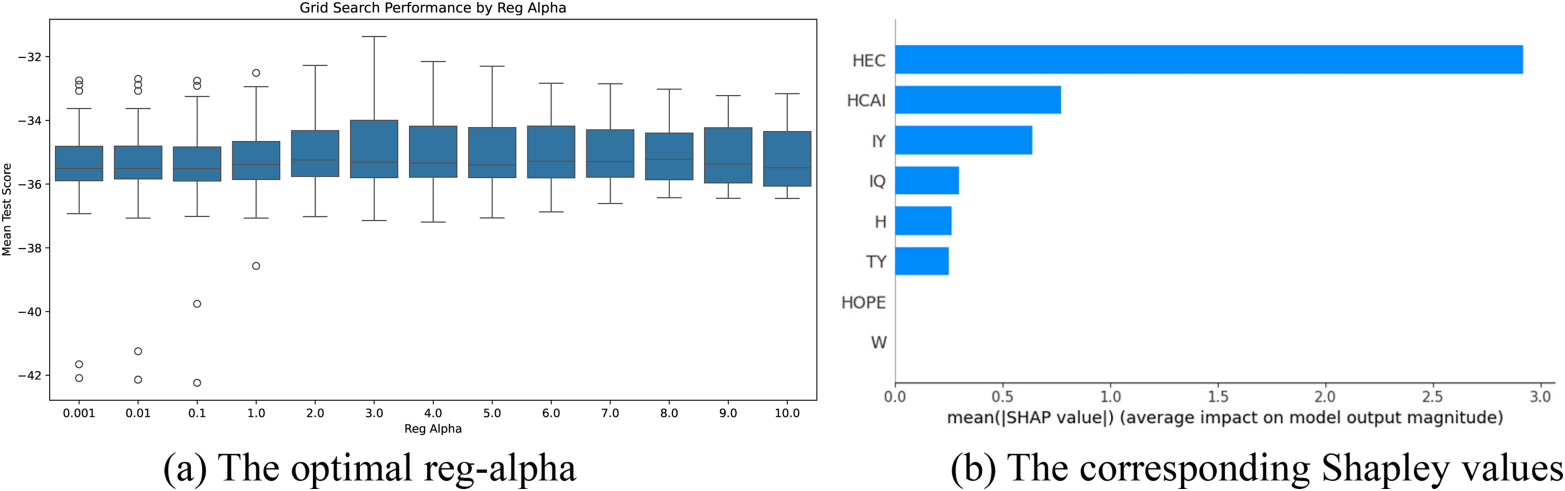
Visualization of screening results for the normal-weight bat intervention of the AA group. **(a)** The optimal reg-alpha. **(b)** The corresponding shapley values.

**Figure 11:**
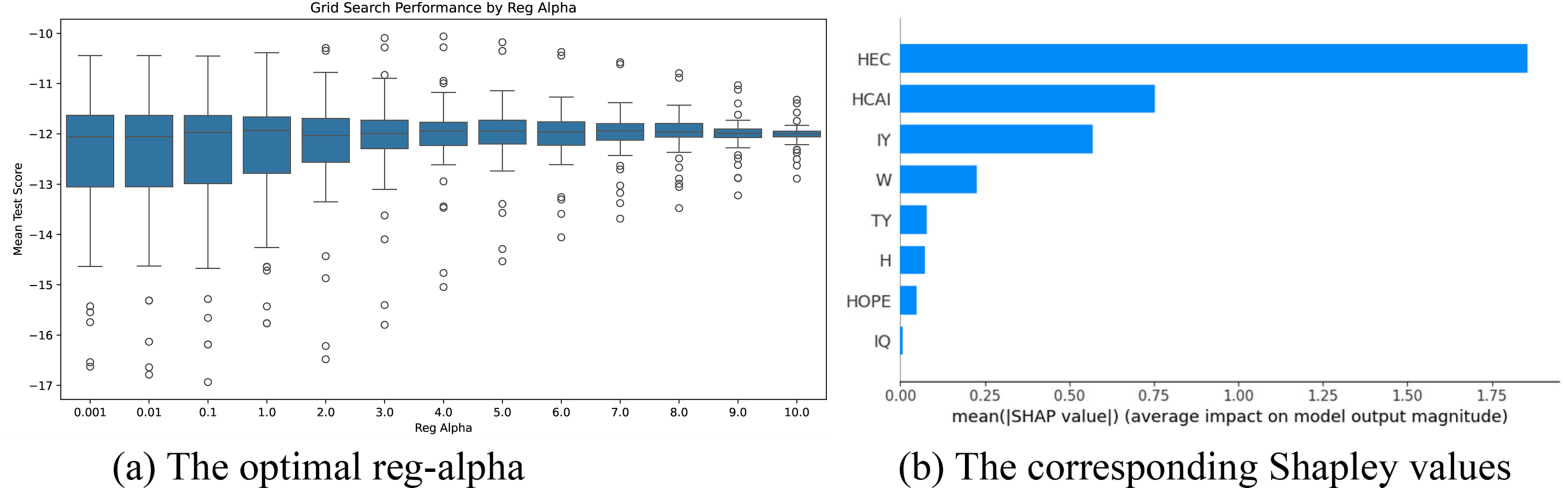
Visualization of screening results for the weight bat intervention of the AA group. **(a)** The optimal reg-alpha. **(b)** The corresponding shapley values.

**Figure 12:**
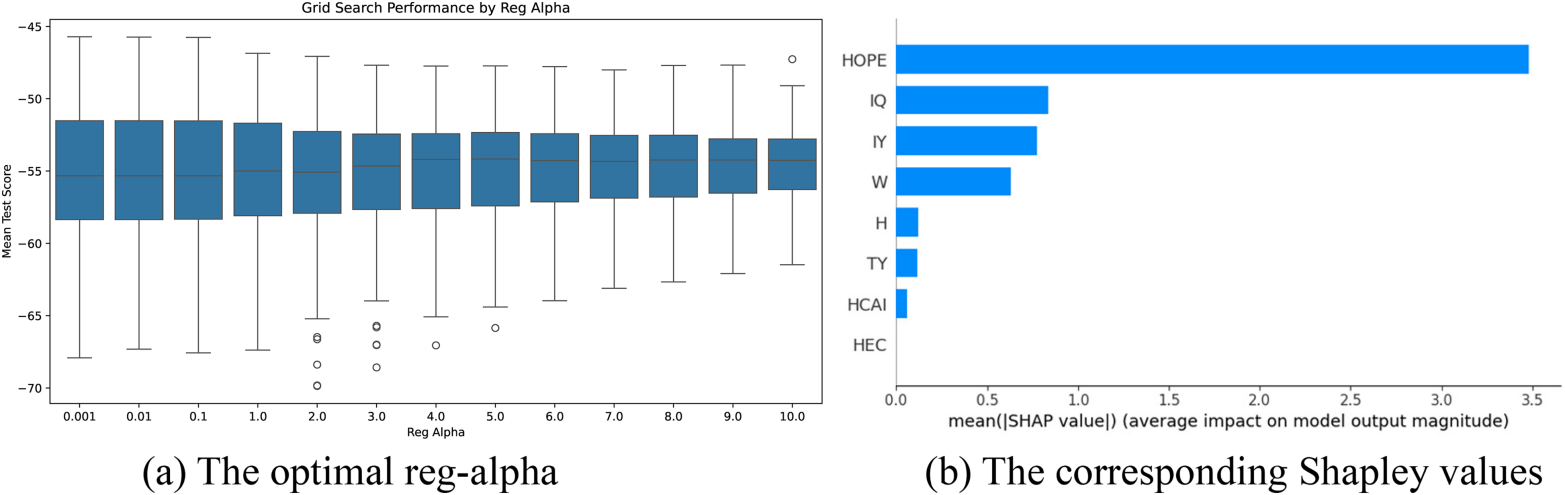
Visualization of screening results for the reduced-weight bat intervention of the AA group. **(a)** The optimal reg-alpha. **(b)** The corresponding shapley values.

The optimal parameter tuning results for reg_alpha in the attack angle intervention factor selection models based on the XGBoost algorithm are shown in Figures 10a–12a. Optimal mean test scores (MTS) for Models 4–6 were obtained when the reg_alpha parameters in these models were assigned the values 3, 1, and 4, respectively. The ranking results of the corresponding Sharply values are depicted in Figures 10b–12b. These illustrations show that the core indicators IY and IQ impacted the post-test attack angles of the intervention groups that used normal-weight bats (Figure 10b); IY affected the post-test attack angles of the intervention groups that used weight bats (Figure 11b); and IQ and IY impacted the post-test batting of the intervention groups that used reduced-weight bats (Figure 12b). As shown in Figures 7b–12b, IBS, TY, H, and W exhibited significant correlations with bat speed, aligning with Szymanski et al.'s (13) earlier findings.

## 4 Discussion

This study evaluated the effects of dry swing intervention on the bat speed and stability in athletes across age groups through a comparative analysis of kinematic data and intervention factors. The study aimed to confirm how dry swing intervention affects the kinematic characteristics of bat speed and stability among athletes in different age groups and to identify the underlying factors contributing to these differences. Ultimately, this study seeks to provide theoretical support for the scientific training and guidance of athletes in various age groups.

The findings of this experimental study are as follows. Among athletes under 18 years of age, dry swing intervention using weight bats significantly changed the range of internal and external rotations of the hips. For those in the 14–15 year age range, dry swing intervention using weight bats led to significant performance changes. Regarding athletes under 18 years of age, particularly those with less than 9.5 years of training term, Tsutsui (19) found a positive correlation between hip internal rotation and bat speed. Additionally, Tsutsui (20) observed that, due to adolescents' generally weaker lower limb strength, energy accumulated through hip internal rotation during the preparatory phase provides momentum for subsequent rotation. This study found that for the 14–15 age group, the intervention group using weight bats exhibited a significant difference in the LHER indicator (*p* = 0.045, ES = 1.619). Furthermore, the LHIR and BS indicator demonstrated a substantial effect size (ES = 0.727; ES = 0.503), and both the LHIR and LHER indicators for athletes aged 14–15 years exhibited improvements, as evidenced by the post-test results. These findings likely stem from the athletes' relative youth: limited strength and underdeveloped movement patterns constrain their ability to handle a heavier bat effectively, leading them to rely more on hip internal rotation to accumulate energy. Furthermore, their shorter training durations and still-maturing neuromuscular systems result in less fixed movement patterns, making them more susceptible to changes in bat weight—especially as they transition from soft to hard baseballs. In contrast, university and professional athletes—with extensive training durations—demonstrated smaller pre- and post- test changes in bat speed. However, none of the intervention groups showed a significant difference in pre- and post-test bat speed, which aligns with previous studies (6, 7). Nonetheless, this study indicates that short-term dry swing intervention with a bat differing from one's usual bat (either heavier or lighter) can significantly alter hip internal and external rotation in adolescent athletes and affect their bat speed. Adolescents thus appear more sensitive to changes in bat weight, which can trigger a reorganization of batting movement patterns in response to external conditions. Compared with the prior research (4–7), applying effect sizes (ES) provides a more refined assessment of intervention effectiveness. In the adolescent group of this study, changes in bat speed due to dry swing intervention were partially consistent with, yet also differed from, previous findings—possibly due to sample selection (adolescents vs. adults) or the choice of intervention tools. Future studies should incorporate actual-game contexts—considering opponents' strategies and whole-body coordination indicators—to further evaluate the comprehensive impact of dry swing intervention on adolescent game performance.

**Figure 13:**
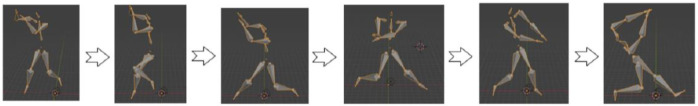
A full-body motion-capture Skeleton was created from cloud voxels, which quantify Athletes’ kinematic parameters.

This study identified significant differences in attack angles among the normal-weight bat intervention groups of the university student group and the 12–14 age group (*p* = 0.018, ES = 0.456; *p* = 0.027, ES = −0.315). This finding contradicts Williams' et al. (6), they suggestion that when attack angles approach zero, the bat aligns with the horizontal plane of the ball, thereby enhancing batting quality and speed. Through multiple batting experiments and a combined analysis of kinematic data and skeletal images from point clouds (Figure 13), this study found that during the batting process, as the attack angle increases, the ball's horizontal plane is positioned higher than the bat's motion trajectory at the moment of impact. In this scenario, the bat is more likely to strike the lower part of the ball, resulting in a fly ball. Furthermore, from the perspective of athletes' physical development, changes in attack angles are influenced by biomechanical factors. For instance, adolescent athletes often have underdeveloped muscle strength and suboptimal neuronal recruitment capabilities, which limit their stability and self-control. These factors contribute to the differences observed in attack angles, particularly among juniors.

The current experimental study demonstrated that, with the exception of the softball group (ages 12–14), dry swing intervention using normal-weight bats exhibited minimal acute effects on batting stability. Comparative analysis of attack angle effect sizes among hardball-trained athletes revealed that the normal-weight intervention group showed the smallest absolute effect size values (closest to zero) between pre- and post-intervention measurements. This result indicates that regularly used bats have the least acute impact on batting stability, aiding athletes in better controlling the bat during actual batting. Notably, that adolescents' higher adaptability to external stimuli (such as changes in bat weight and pitcher tempo) means they can also exhibit altered movement patterns more easily in the short term. This underscores the importance of choosing appropriate training equipment for different age groups, in order to enhance batting efficiency while minimizing disruptions to batting stability. Additionally, the SHAP-based analysis corresponding to the experimental results shows that the tilt and elevation angles during dry swing intervention significantly influence the attack angle in post-tests. Studies by Iencean et al. (21) and Cholewicki et al. (22) have confirmed that the stability of localized spinal muscle groups is fundamental to trunk rotation. Using heavier or lighter bats alters the spinal “trunk rotation kinetic chain”, affecting both stability and trunk rotation speed during batting. Therefore, pre-game warm-up swings with infrequently used bats negatively impacts stability during actual batting.

The results of a comparative analysis of the reduced-weight bat intervention groups across age brackets indicate that the reduced-weight bat intervention had almost no impact on bat speed in all groups (ES = 0.002). Both normal-weight and weight bat interventions exhibited declines in post-test bat speed (ES = −0.055 and ES = −0.100, respectively). The intervention feature results selected by the model reveal that the core indicators IPHER and IPHMS in the normal-weight bat intervention groups significantly influenced post-test bat speed. Contrastingly, the core indicators ILHIR, IL3IR, and IL3ER in the weight bat intervention groups significantly impacted post-test bat speed. Previous research has shown that when bat speed decreases, the bat's MOI increases (23). The MOI of normal-weight and weight bats is smaller than that of reduced-weight bats, indicating that athletes require greater rotational torque to execute the swing, which can easily spur core muscle fatigue and reduced swing power output. Biomechanically, swinging a weighted bat has distinct effects on proximal and distal muscle groups (3). Gray (3) highlighted the rotational kinetic energy transferred from the pelvis to the torso and spine as a critical factor in maximizing bat speed. The muscle endurance, maximal strength, and explosive power of the proximal and distal segments contribute to the collective morphology, which is necessary to maximize rotational speed (24). As part of the lumbar spine, L3 is an important proximal bodily segment. In the weighted bat intervention groups, IL3IR and IL3ER significantly affected post-test bat speed, indicating that the impact of the weight bat unbalances the proximal and distal morphologies, pinpointing the biomechanical cause of the decline in bat speed evidenced by the post-test. Furthermore, such an imbalance is likely caused by accumulated muscle fatigue, particularly owing to repetitive interventions, which easily interrupt the coordination between proximal and distal muscle groups, affecting bat speed.

Contrary to the viewpoints in Bassett et al. (10), this study advises against the usage of weight bats during warm-up swings, particularly for the adolescent athletes. The rationale analyzing is interpreted through the lens of both biomechanics and psychology. Biomechanically, first, during actual batting, the spine seldom locates true neutral alignment (14), rotational forces inevitably incur axial eccentric elongation and spinal rotation. Increased bat weight exacerbates unilateral loading, resulting in alterations in tilt and elevation angles. This amplification of pre-existing axial eccentric elongation stretches ipsilateral erector spinae muscles, compromising spinal stability (8). Second, adolescent athletes, who are in a critical phase of neuromuscular system development (25, 30), may experience more neuromuscular adaptation stress (5) when exposed to the increased muscular demands and movement complexity associated with weighted bat swing. This adaptive response manifests not only as localized muscle fatigue, but also disrupts the refinement of swing and movement pattern consolidation in developing athletes, ultimately compromising movement fluency and kinetic transfer efficiency. Psychologically, the junior athletes demonstrate more sensitivity to error-related anxiety (26, 27). Under the actual batting scenario, the weighted bats may undermine batting confidence and incur performance-inhibiting apprehension, consequently degrading batting quality. Finally, Williams et al. (6) identified fatigue accumulation as a potential modulator of bat speed variation, a finding in Miller et al. (28) also demonstrated correlations between bat speed variations and fatigue accumulation. The findings in this study show that bat-weighting dry swing intervention predominantly yield negative effects in adolescent athletes, manifesting as increased muscular fatigue and compromised bat speed and stability. Thus the coaches should exercise judicious caution when performing warm-up swings with weighted bat, especially for the adolescent athletes.

Additionally, although a few factors reached statistical significance (e.g., bating tilt angle in 12–14-year-olds normal-bat group: *p* = 0.047), their trivial effect sizes (ES = 0.173) warrant caution. As our primary outcomes were bat speed and attack angle changes- with other bat and body kinematics being exploratory—we prioritized sensitivity over multiple comparison corrections. Readers should interpret the secondary outcomes through the lens of effect sizes and biomechanical relevance.

## 5 Conclusion and limitation

### 5.1 Conclusion

Findings highlight age-dependent variations in athlete responses to dry swing intervention, impacted by biomechanical profiles, training term, and bat weight adaptability. Acute pre-dry swing intervention with weight, normal-weight, or reduced-weight bat demonstrated no significantly effects on subsequent in-game bat speed. However, a dry swing intervention using a weight bat has a more pronounced negative effect on bat speed than interventions using a reduced-weight or normal-weight bat. Moreover, compared to normal-weight bats, weight and reduced-weight bats exert greater impacts on attack angles. Given these results, athletes with shorter training term—especially adolescents—should avoid using weight or reduced-weight bats during pre-game warm-up swings. Excessive use of these bats may disrupt the kinetic chain involved in batting and compromise spinal stability in young athletes. Instead of helping young athletes establish optimal force production patterns, these practices may increase the risk of injury. Thus, dry swing intervention for athletes across age groups should be conducted based on their individual characteristics, with personalized instructions provided to achieve the best training outcomes.

### 5.2 Limitation

Despite the relatively significant theoretical and practical contributions of this study, the authors acknowledge several limitations that may affect the validity and generalizability of the findings. First, the small sample size may limit the strength of the conclusions drawn. When interpreting secondary outcomes, results showing statistical significance but small effect sizes warrant caution; replicating them in future studies with larger samples or more rigorous designs would enhance their credibility and external validity. Next, the homogeneity of the participant pool, primarily consisting of athletes with similar training backgrounds, restricts the applicability of the results to a broader and more diverse athletic population. Then, baseball is a niche sport in China, resulting in considerable variability in training backgrounds among athletes. This variability may lead to differences in responses to bat weight interventions, potentially affecting the generalizability of the study's findings. Finally, conducting the study within a single geographical location may limit the applicability of the results to athletes from different regions with varying training environments and cultural backgrounds. Future research should aim to address these limitations by incorporating larger and more diverse sample sizes, integrating dry swing intervention with visually guided modules, utilizing a wider range of equipment such as motion capture and analytical methods such as motion modeling to enhance the robustness and generalizability of the findings.

## 6 Practical applications

The findings of this study merit several factors that may directly impact coaches and athletes during warm-up swings to optimize actual batting. Normal-weight bats appear more suitable for adolescent or less experienced baseball athletes, as they generally do not disrupt swing patterns while reducing neuromuscular fatigue risks, thereby maintaining optimal psychological readiness during actual batting. Although some exist studies (10, 27) suggests that adult athletes may select bats based on subjective comfort during warm-up swings, this study thinks that coaches should carefully evaluate multiple factors—including strength-to-mass ratio, technical proficiency, and injury history—when considering the implementation of weighted or non-standard bats for adult or elite-level athletes, to prevent potential adverse effects. Furthermore, the findings highlight the biomechanical primacy of hip kinematics in governing both bat speed and stability. Consequently, targeted strength training focusing on hip joint musculature should be implemented to enhance batting performance while mitigating injury risks.

## Data Availability

The original contributions presented in the study are included in the article/Supplementary Material, further inquiries can be directed to the corresponding author.

## References

[1] Welch CM Banks SA Cook FF Draovitch P. Hitting a baseball: a biomechanical description. J Orthop Sports Phys Ther. (1995) 22(5):193–201. 10.2519/jospt.1995.22.5.193 8580946

[2] Szymanski DJ Renne C Spaniol FJ. Contributing factors for increased bat swing velocity. J Strength Cond Res. (2009) 23(4):1338–52. 10.1519/JSC.0b013e318194e09c 19528868

[3] Gray R. How do batters use visual, auditory, and tactile information about the success of a baseball swing? Res Q Exerc Sport. (2009) 80(3):491–501. 10.1080/02701367.2009.10599587 19791635

[4] Montoya BS Brown LE Coburn JW Zinder SM. Effect of warm-up with different weighted bats on normal baseball bat velocity. J Strength Cond Res. (2009) 23(5):1566–9. 10.1519/JSC.0b013e3181a3929e 19593220

[5] Southard D Groomer L. Warm-up with baseball bats of varying moments of inertia: effect on bat velocity and swing pattern. Res Q Exerc Sport. (2003) 74:270–6. 10.1080/02701367.2003.10609091 14510291

[6] Williams CC Gdovin JR Wilson SJ Cazas-Moreno VL Eason JD Hoke EL The effects of various weighted implements on baseball swing kinematics in collegiate baseball players. J Strength Cond Res. (2019) 33(5):1347–53. 10.1519/JSC.0000000000002020 29019867

[7] Wilson JM Miller AL Szymanski DJ Duncan NM Andersen JC Alcantara ZG Effects of various warm-up devices and rest period lengths on batting velocity and acceleration of intercollegiate baseball players. J Strength Cond Res. (2012) 26(9):2317–23. 10.1519/JSC.0b013e31823daebf 22037096

[8] McGill SM McDermott ART Fenwick CMJ. Comparison of different strongman events: trunk muscle activation and lumbar spine motion, load, and stiffness. J Strength Cond Res. (2009) 23(4):1148–61. 10.1519/JSC.0b013e318198f8f7 19528856

[9] Palmer TG Uhl TL. Interday reliability of peak muscular power outputs on an isotonic dynamometer and assessment of active trunk control using the chop and lift tests. J Athl Train. (2011) 46(2):150–9. 10.4085/1062-6050-46.2.150 21391800 PMC3070502

[10] Bassett KE Szymanski DJ Beiser EJ Till ME Medlin GL DeRenne C. Effects of various warm-up devices on bat swing velocity of college softball players. J Strength Cond Res. (2011) 25:S71. 10.1097/01.JSC.0000395692.98600.f1 22201694

[11] Palmer TG McCabe M. The effect of a novel weight-supported kinetic chain resistance training program on proximal core muscular endurance, trunk-to-arm muscular power, and bat swing speed. J Strength Cond Res. (2023) 37(11):2130–40. 10.1519/JSC.0000000000004520 37883393

[12] Reyes GC Dickin DC Dolny DG Crusat NJ. Effects of muscular strength, exercise order, and acute whole-body vibration exposure on bat swing speed. J Strength Cond Res. (2010) 24(12):3234–40. 10.1519/JSC.0b013e3181e727a2 21088545

[13] Szymanski DJ Beiser EJ Bassett KE Till ME. Relationships between sports performance variables and bat swing velocity of collegiate baseball players. J Strength Cond Res. (2011) 25:S122. 10.1097/01.JSC.0000395775.89191.33 21240027

[14] Rahnama L Acik C Dy C Keslacy S. Unilateral baseball pitching: morphological and functional adaptations in the neck muscles. Front Sports Act Living. (2025) 7:1452412. 10.3389/fspor.2025.1452412 39995574 PMC11847819

[15] Oytun M Tinazci C Sekeroglu B Acikada C Yavuz HU. Performance prediction and evaluation in female handball players using machine learning models. IEEE Access. (2020) 8:116321–35. 10.1109/ACCESS.2020.3004182

[16] Rommers N Rössler R Verhagen E Vandecasteele F Verstockt S Vaeyens R A machine learning approach to assess injury risk in elite youth football players. Med Sci Sports Exercise. (2020) 52(8):1745–51. 10.1249/MSS.0000000000002305 32079917

[17] Downs JL Bordelon NM Friesen KB Shannon DM Oliver GD. Kinematic differences exist between the fastball, changeup, curveball, and dropball pitch types in collegiate softball pitchers. Am J Sports Med. (2021) 49(4):1065–72. 10.1177/0363546520988172 33606550

[18] Faul F Erdfelder E Lang A-G Buchner A. G* power 3: a flexible statistical power analysis program for the social, behavioral, and biomedical sciences. Behav Res Methods. (2007) 39(2):175–91. 10.3758/BF03193146 17695343

[19] Tsutsui T Maemichi T Torii S. Identification of physical characteristics associated with swing velocity of batting in youth baseball players. J Sports Med Phys Fitness. (2021) 62(8):1029–36. 10.23736/s0022-4707.21.12500-9 34028244

[20] Tsutsui T Sakata J Sakamaki W Maemichi T Torii S. Longitudinal changes in youth baseball batting based on body rotation and separation. BMC Sports Sci Med Rehabil. (2023) 15(1):162. 10.1186/s13102-023-00774-5 38017563 PMC10683358

[21] Iencean SM. Classification of spinal injuries based on the essential traumatic spinal mechanisms. Spinal Cord. (2003) 41(7):385–96. 10.1038/sj.sc.3101468 12815370

[22] Cholewicki J Simons APD Radebold A. Effects of external trunk loads on lumbar spine stability. J Biomech. (2000) 33(11):1377–85. 10.1016/S0021-9290(00)00118-4 10940396

[23] Koenig K Mitchell ND Hannigan TE Clutter JK. The influence of moment of inertia on baseball/softball bat swing speed. Sports Eng. (2004) 7:105–17. 10.1007/BF02915922

[24] Smith L Kensrud J. Field and laboratory measurements of softball player swing speed and bat performance. Sports Eng. (2014) 17:75–82. 10.1007/s12283-013-0126-y

[25] Malina RM Rogol AD Cumming SP Coelho e Silva MJ Figueiredo AJ. Biological maturation of youth athletes: assessment and implications. Br J Sports Med. (2015) 49(13):852–9. 10.1136/bjsports-2015-094623 26084525

[26] Conroy DE Coatsworth JD Kaye MP. Consistency of fear of failure score meanings among 8-to 18-year-old female athletes. Educ Psychol Meas. (2007) 67(2):300–10. 10.1177/0013164406288174

[27] Sagar SS Lavallee D. The developmental origins of fear of failure in adolescent athletes: examining parental practices. Psychol Sport Exerc. (2010) 11(3):177–87. 10.1016/j.psychsport.2010.01.004

[28] Miller RM Heishman AD Freitas EDS Bemben MG. Evaluating the effects of underloaded and overloaded warm-ups on subsequent swing velocity. J Strength Cond Res. (2020) 34(4):1071–7. 10.1519/JSC.0000000000002206 32205835

[29] Glebova E Madsen DØ Mihaľová P Géczi G Mittelman A Jorgič B. Artificial intelligence development and dissemination impact on the sports industry labor market. Front Sports Act Living. (2024) 6:1363892. 10.3389/fspor.2024.1363892 38606117 PMC11007172

[30] Williams MD Ramirez-Campillo R Chaabene H Moran J. Neuromuscular training and motor control in youth athletes: a meta-analysis. Percept Mot Skills. (2021) 128(5):1975–97. 10.1177/00315125211029006 34293993 PMC8414837

[31] Punchihewa NG Miyazaki S Chosa E Yamako G. Efficacy of inertial measurement units in the evaluation of trunk and hand kinematics in baseball hitting. Sensors. (2020) 20(24):7331. 10.3390/s20247331 33419341 PMC7766213

[32] Kang M Ragan BG Park JH. Issues in outcomes research: an overview of randomization techniques for clinical trials. J Athl Train. (2008) 43(2):215–21. 10.4085/1062-6050-43.2.215 18345348 PMC2267325

